# A Rare Complication of Diaphragm Plication: Acute Liver Injury From Hepatic Compartment Syndrome

**DOI:** 10.14309/crj.0000000000001127

**Published:** 2023-08-30

**Authors:** Michael Chang, Samantha Menegas, David Uihwan Lee, Muhammad Baraa Hammami, Sasan Sakiani

**Affiliations:** 1 University of Maryland School of Medicine, Baltimore, MD; 2 Division of Gastroenterology and Hepatology, Department of Medicine, University of Maryland School of Medicine, Baltimore, MD

**Keywords:** diaphragm plication, acute liver injury, liver, hepatic compartment syndrome, surgery

## Abstract

Diaphragm plication is a surgical treatment of unilateral diaphragm paralysis, in which the affected diaphragm is sutured in place. Because the right diaphragm sits on top of the liver, right-sided diaphragm plication can injure the liver and lead to hepatic compartment syndrome resulting in acute liver injury. We report a case of a 59-year-old woman with a history of multilevel disk degeneration and alcohol use disorder who underwent right-sided diaphragm plication. After surgery, she complained of abdominal pain and was found to have severely elevated liver-associated enzymes and evidence of acute liver injury, which resolved with supportive care.

## INTRODUCTION

Diaphragm plication is a procedure that can be used in the management of unilateral diaphragm paralysis causing significant dyspnea.^1,2^ This procedure involves creating folds in the affected diaphragm and suturing it in place. This improves the expansion and gas exchange of the ipsilateral lung.^3^ Some complications of diaphragmatic plication include pneumonia, pulmonary embolism, and respiratory failure.^4^ This procedure has typically been performed through standard thoracotomy, although video-assisted thoracoscopic surgery (VATS) is gaining popularity as a less invasive alternative.^4^

Hepatic compartment syndrome (HCS) is a phenomenon in which elevated intraparenchymal hepatic pressure leads to acute liver injury. This is conceptually similar to abdominal compartment syndrome, but occurs within the liver parenchyma. Prior case reports have described HCS in the context of subcapsular and/or intrahepatic hematomas. These resulted from trauma^5^ or procedures such as liver biopsy or cholecystostomy.^6–9^ There is 1 case report of HCS occurring secondary to right-sided diaphragm plication.^10^ In this clinical scenario, we describe a rare case of liver injury arising after a plication procedure.

## CASE REPORT

A 59-year-old woman with a history of multilevel disk degeneration, alcohol use disorder, and gastric bypass presented for scheduled surgery for an elevated right hemidiaphragm. Several months earlier, she had been diagnosed with alcoholic and nonalcoholic fatty liver disease by ambulatory ultrasound, which showed steatosis and hepatomegaly with a diameter of 20.5 cm. She had complained of dyspnea 4 months before and had a normal chest computed tomography angiography and echocardiogram at the time. A sniff test later revealed the absence of any right diaphragmatic excursion, which is consistent with right-sided nonspecific phrenic nerve palsy. Magnetic resonance imaging of the cervical spine showed multilevel degenerative facet arthropathy, particularly between C3 and C6 vertebrae. Right-sided VATS plication of the diaphragm was performed by 3 port sites (1 in the midaxillary line at the sixth intercostal space, 1 more in the same intercostal space, and 1 in the midclavicular line at the fourth intercostal space). Multiple layers of plication were performed without complications or blood loss, and the patient was stable when transitioned to the postanesthesia care unit.

**Figure 1.**
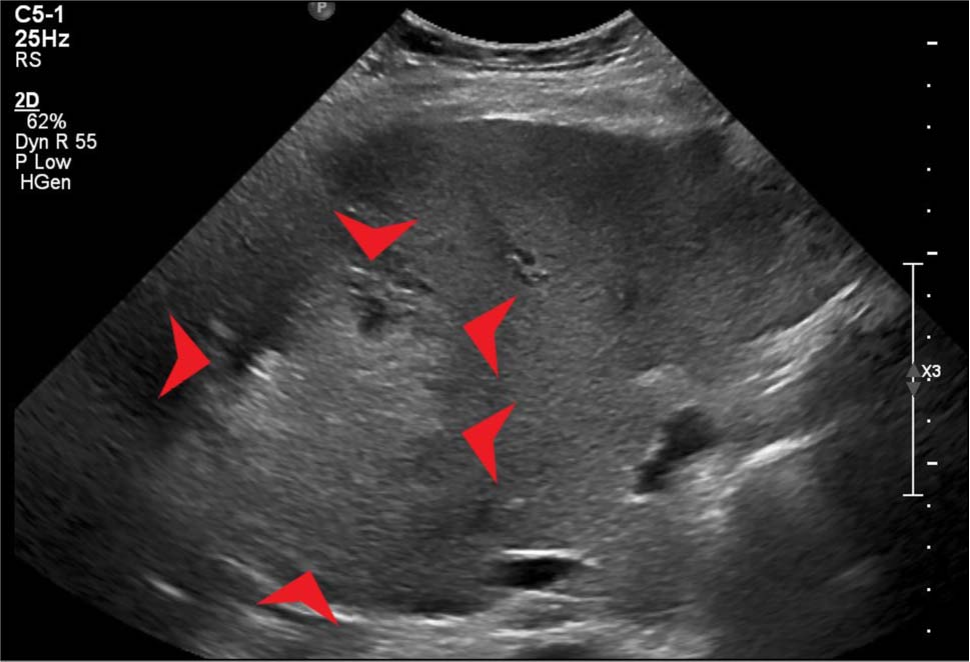
Arrows outline an ill-defined, heterogeneous, predominantly hyperechoic lesion in the peripheral right liver.

Two days after surgery, the patient expressed abdominal discomfort in the epigastric to the right upper quadrant area and was confused overnight. Laboratory evaluation revealed elevations in her liver-associated enzymes (LAEs), which included an aspartate aminotransferase (AST) level of 4,099 U/L, alanine aminotransferase (ALT) level of 2,303 U/L, total bilirubin concentration of 1.4 mg/dL, alkaline phosphatase concentration of 324 U/L, and international normalized ratio of 1.4. The workup for the viral hepatitis panel was unremarkable for acute hepatitis A, B, or C infection. In addition, the patient's vitals had remained stable throughout her hospitalization without any episodes of hypotension, and the patient had not received any potential hepatotoxic medications. Liver-specific ultrasonography was acquired to visualize the liver parenchyma and hepatic vessels. This demonstrated a heterogeneous, ill-defined lesion in the right liver measuring 10.1 × 5.7 × 5.6 cm. Per radiologic interpretation, it was believed that the lesion most likely represented after surgical bruising or ischemia/infarction (Figure 1). The hepatic vasculature was patent. The flow in the main portal vein was antegrade and biphasic, and the flow in the 3 hepatic veins was triphasic and predominantly antegrade. The hepatic artery (HA) peak systolic velocity was 117 cm/s, and velocities of the portal vein, hepatic vein, and HA were within the normal range—15–40 cm/s, <20 cm/s, and 55 ± 12 cm/s, respectively. Her AST and ALT continued to downtrend from 1,346 U/L and 1,695 U/L, respectively, on postoperative day 3 to 81 U/L and 375 U/L, respectively, on postoperative day 6 with daily improvements in mentation. At this point, her abdominal pain had resolved.

## DISCUSSION

Our patient underwent a right-sided plication procedure for diaphragmatic paralysis and subsequently had elevations in LAEs, specifically in AST and ALT. Although other etiologies of liver injury were considered, there was no laboratory evidence of viral hepatitis, and the medication list did not obviate a pharmacologic source of hepatic injury. In addition, the patient's vitals had remained stable throughout her hospital stay with no documentation of intraoperative hypotension or vasopressor requirements, making ischemic hepatitis and hemodynamic instability unlikely. Although the patient had a history of alcohol use disorder, acute alcoholic hepatitis was believed to be unlikely given AST/ALT levels greater than 400 IU/L and lack of worsening of jaundice. Her bilirubin, international normalized ratio, and improving mentation revealed acute liver injury without liver failure. In considering the differentials, the plication procedure may have elicited an irritation of the hepatic capsule, which sits below the diaphragmatic fold. In addition to blunt irritation, there may have been a distortion of the hepatic vessels that trespass the liver and capsule, leading to an area of ischemia observed in liver-focused ultrasonography. The patent hepatic vessels visualized in Doppler topography suggest the ischemic injury to be independent of stenotic or thrombotic causes.

The patient also had an underlying liver disorder and hepatomegaly, which likely made her more susceptible to plication-related hepatic injury. Her liver was enlarged at 20.5 cm, leading to a greater likelihood of causing force trauma and irritation of the hepatic walls during plication surgery and changes in abdominal pressure from a repaired diaphragm. Although current literature does not differentiate between patients with hepatomegaly when considering open thoracotomy vs VATS, some studies suggest a benefit of performing open thoracotomy over VATS given the upward movement of the right hemidiaphragm.^11,12^ Fortunately, our patient improved with symptomatic management after surgery, which is the standard treatment of hepatic traumas in hemodynamically stable patients.^5^

Our case represents the second report of HCS occurring after a right-sided diaphragm plication.^10^ However, unlike the prior report, our patient underwent VATS as opposed to open thoracotomy. VATS plication is believed to be associated with fewer complications than open thoracotomy, but our case shows that hepatic injury may still occur with VATS plication. The modes of injury may mechanistically differ depending on the type of operation performed. With open thoracotomy plication, there may be a larger area of diaphragmatic involvement and a possibility of hematoma forming around the outgoing hepatic vessels. By contrast, VATS plication is more likely to be localized and hence develops controlled areas of hematoma, possibly affecting vessels only in close proximity.

It is important to consider the possibility of postplication liver injury among susceptible patients with enlarged livers. Those who undergo a right-sided plication procedure and develop abdominal pain with elevations in LAEs should warrant a clinical workup that includes a radiographic examination of the liver parenchyma and vasculature to rule out the possibility of postplication liver injury. If diagnostically confirmed, LAEs should be monitored until near resolution to indicate hepatic recovery.

## DISCLOSURES

Author contributions: M. Chang and S. Menegas: study concept and design, literature search, analysis and interpretation of data, and drafting of the manuscript. D. Lee and MB Hammami: study supervision. D. Lee, MB Hammami, and S. Sakiani: critical revision of the manuscript for important intellectual content. MB Hammami is the article guarantor.

Financial disclosure: None to report.

Informed consent was obtained for this case report.
